# A cascaded interferometer-microresonator structure for photonic reservoir computing

**DOI:** 10.1038/s41598-026-39410-w

**Published:** 2026-02-14

**Authors:** Amideddin Mataji-Kojouri, Sebastian Kühl, Mohammad Seifi Laleh, Chandan Upadhyay, Stephan Pachnicke, Kambiz Jamshidi

**Affiliations:** 1 Integrated Photonic Devices Group, Chair of RF and Photonics Engineering, TU Dresden, Helmholzstr. 18, 01069 Dresden, Germany; 2 Chair of Communications, Kiel University, Kaiserstr. 2, 24143 Kiel, Germany

**Keywords:** Optical computing, Reservoir computing, Photonic integrated circuits, Silicon microring resonators, Photonic signal equalization, Engineering, Optics and photonics, Physics

## Abstract

Complex dynamics of silicon microring resonators fed by delay elements enable high-speed photonic reservoir computing. Implementing delay elements on a photonic chip can be challenging when the required delay should match the time scales of nonlinear effects in silicon. To increase the computation speed and preclude any need for very long delay lines and silicon’s nonlinear effects, we opt for a delay-based photonic reservoir where the nonlinear functionality is provided merely by either of amplitude or phase modulations along with photodetection. Our simulations show that by supplementing its memory with digital memory in the electronic output layer, the time-delay photonic reservoir composed of a Mach-Zehnder interferometer and a microring resonator can perform computations almost one order of magnitude faster than those based on silicon’s nonlinearity with its speed only limited by the modulation/detection bandwidth. We also show that such a time-delay reservoir can perform accurately in NARMA-10, Mackey-Glass, and Santa-Fe prediction tasks (normalized mean-squared error, NMSE in the range 0.002–0.05), and enables signal equalization in optical communication systems.

**Supplementary Information:**

The online version contains supplementary material available at 10.1038/s41598-026-39410-w.

## Introduction

Photonic reservoir computers employ complex dynamics of a photonic system to process data and perform computational tasks at speeds readily exceeding 1 GHz^1–3^. The reservoir is a randomly connected network with static weights that is fed by an external signal at its input layer. The output layer is trained to produce the desired output by reading the state of the reservoir. Computation with these systems is enabled by their complex dynamics that can be described by nonlinear delay differential equations^4,5^. Due to their rich nonlinear dynamics^6,7^ and relatively simple implementation, silicon microring resonators (MRRs) have been widely investigated for implementing photonic reservoir computers^8–13^ Nonlinear phenomena in a silicon MRR include two-photon absorption (TPA), which generates free carriers that subsequently contribute to free-carrier absorption (FCA) and free-carrier dispersion (FCD), as well as thermo-optic (TO) effect and on the intrinsic optical Kerr effect of silicon. These nonlinear optical phenomena occur in different time scales. The fastest is the optical Kerr effect which is almost instantaneous due to its electronic origin. FCD and FCA exhibit intermediate response times on the order of a few nanoseconds determined by the carrier lifetime. The TO effect demonstrates the slowest response dominated by the heat diffusion processes, with characteristic times ranging from tens to hundreds of nanoseconds^14,15^. Although the Kerr effect is present in silicon MRRs, it is usually overshadowed by the relatively slower but significantly stronger FCD/FCA effects which are the dominant nonlinear mechanisms in a silicon MRR. To obtain a faster response, it is possible to decrease the free carrier lifetime by applying a reverse bias, which facilitates carrier extraction. However, faster decay also weakens the FCD/FCA processes and increases their corresponding threshold power, thus limiting the performance of the reservoir. Silicon MRRs can be also loaded by delayed feedback to exhibit a better performance in computing compared to a single MRR. Free-carrier nonlinearity has been extensively studied for reservoir computing^16–19^. In time-delay reservoir computers, complex feedback elements are employed to provide a delay that matches the free carrier lifetime (a few nanoseconds)^10,11,13^. Bit durations are also comparable to free-carrier lifetime to efficiently employ the FCD/FCA nonlinearity for computation. It is found that in such configurations, FCD/FCA nonlinearity does not efficiently improve the performance for certain computational tasks such as NARMA-10 while it provides improvements for other tasks (*e.g.* Santa-FE)^10,11,13^. To avoid challenges associated with implementing long delay lines, we investigate time-delay photonic reservoir computation using a linear PIC in which nonlinearity is provided at the modulation and detection steps. Reservoir computing has been proposed for time series prediction using different architectures, such as non-fading memory and recurrent resonator neural networks^17,18^, swirl-topology-based architecture of MRRs^20^, single microring resonators operated at their self-pulsing regime^17^, and time-delay reservoir computers. In this work, we employ a time-delay architecture in which the masking process is an important factor that influences computation speed. We investigate binary masking sequences of different lengths to optimize the computation speed and accuracy.

**Fig. 1:**
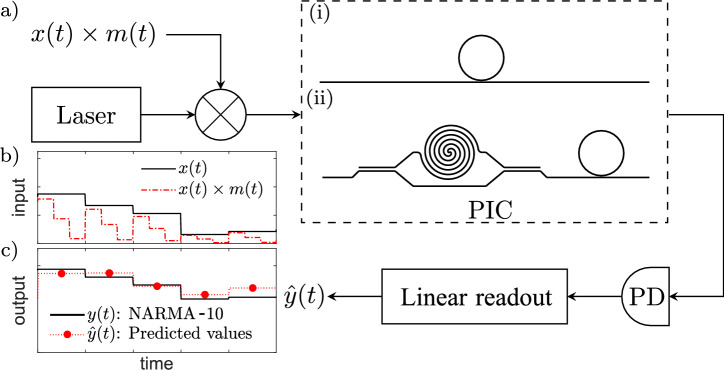
Time-delay photonic reservoir computing. (**a**) A continuous-wave laser light is modulated by a masked input signal and detected using a photodetector (PD) after passing through a photonic integrated circuit (PIC). The photodetected signal is processed at the output layer using an electronic linear readout layer. (**b**) A typical input sequence *x*(*t*) and masked signal $x(t)\times m(t)$ for a mask sequence $M=(1,0.5,0)$. (**c**) Typical desired output signal *y*(*t*) and predicted values ($\hat{y}(t)$). Note the equal symbol duration for the input *x*(*t*) and the predicted output $\hat{y}(t)$.

Following these investigations, we propose a time-delay photonic reservoir composed of an unbalanced Mach-Zehnder interferometer (MZI) along with an all-pass MRR. (Fig. 1). To increase the computation speed we restrict the ring to the linear regime and use a short mask sequence.

The performance of this MZI-MRR-based reservoir is numerically investigated in time-series prediction tasks. Without adding any extra complexity, the proposed reservoir can predict time series at speeds only limited by the modulation speed. Our simulations also show that it can be employed for signal equalization in a short-reach fiber optic transmission system.

The structure of the paper is as follows. In Sect. 2 we summarize the linear and nonlinear models used to describe the behavior of the MRRs. In Sect. 3, we first investigate the impact of nonlinear optical phenomena on the performance of an MRR-based reservoir. Then we study the impact of mask length and output layer dimensionality on the performance of the photonic time-delay reservoir. In Sect. 4 we discuss the performance of the MZI-MRR structure in time series and signal equalization tasks.

## Modeling silicon microring resonators

In this section, we discuss the linear and nonlinear models for predicting the response of an all-pass silicon MRR that is excited through a loss-less coupler. These models can be easily extended to more complicated configurations as will be briefly discussed later. In linear regime, we can use a transfer matrix formalism to find the transfer function for the photonic integrated circuit in the frequency domain. For an all-pass ring resonator, the transfer function is

1 $$\begin{aligned} T(\omega )=\frac{{\sigma -e^{-\gamma \left( \omega \right) l}}}{{1-\sigma e^{-\gamma \left( \omega \right) l}}}, \end{aligned}$$

where *l* is the circumference of the ring resonator, $\gamma \left( \omega \right) =\alpha +jk(\omega )$ is the complex propagation constant, and $\sigma$ is the self-coupling coefficient of the lossless coupler. The frequency dependency of the propagation constant and the coupling coefficients can be calculated using a full-wave modal solver and introduced to the linear model. To calculate the output of the linear ring resonator excited by an input signal in the time domain, we have used the fast Fourier transform (FFT).

To approximate the behavior of the ring resonator in the nonlinear regime, we employ a coupled-mode theory (CMT) analysis^7,14,21,22^. When a silicon ring resonator is excited by an optical input in the near-infrared range, large circulating power in the ring resonator, which is a consequence of the field enhancement at resonance, gives rise to TPA which in turn produces free carriers in the silicon waveguide. The presence of free carriers in silicon increases the loss due to FCA and changes its refractive index due to FCD. Absorption of optical power in the ring can increase the temperature of the resonator and change its refractive index due to the TO effect. Finally, third-order nonlinear susceptibility of silicon (optical Kerr effect) alters the effective index of the waveguide proportional to the circulating light power. The following CMT equations approximate the dynamics of the complex amplitude of the mode in the MRR (*u*), electron-hole pair density (*N*), and temperature change $(\Delta T)$ for an all-pass silicon ring resonator^14,15,22^:

2 $$\begin{aligned} \frac{{du}}{{dt}} & = \left\{ {\frac{{ - j\omega _{{\mathrm{L}}} }}{{n_{0} }}\left[ {\overbrace {{\frac{{n_{2} c_{0} }}{{n_{0} V_{{{\mathrm{Kerr}}}} }}\left| u \right|^{2} }}^{{{\mathrm{Kerr}}}} - \overbrace {{\left( {\sigma _{{{\mathrm{r}}_{{\mathrm{1}}} }} N + \sigma _{{{\mathrm{r}}_{{\mathrm{2}}} }} N^{{0.8}} } \right)}}^{{{\mathrm{FCD}}}} + \overbrace {{\kappa _{\theta } \Delta T}}^{{{\mathrm{TO}}}}} \right]} \right. \\ & \quad \left. { + j\overbrace {{\left( {\omega _{0} - \omega _{{\mathrm{L}}} } \right)}}^{{{\mathrm{detuning}}}} - \frac{{c_{0} }}{{2n_{0} }}\left[ {\overbrace {\alpha }^{{{\mathrm{LL}}}} + \overbrace {{\frac{{\beta _{2} c_{0} }}{{n_{0} V_{{{\mathrm{TPA}}}} }}\left| u \right|^{2} }}^{{{\mathrm{TPA}}}} + \overbrace {{\sigma _{{{\mathrm{FCA}}}} N}}^{{{\mathrm{FCA}}}}} \right]} \right\}u \\ & \quad + \sqrt {\Gamma _{{\mathrm{c}}} } {\mkern 1mu} s_{{{\mathrm{in}}}} \left( t \right), \\ \end{aligned}$$

3 $$\begin{aligned} \frac{d N}{d t} =&\ \frac{c_0^2\beta _2}{n_0^2 2\hbar \omega _\textrm{L} V_{\textrm{FCA}}^2}\left| u\right| ^4- \frac{N}{\tau _{\textrm{car}}}, \end{aligned}$$

4 $$\begin{aligned} \frac{d \Delta T}{d t} =&\ \frac{\left| u \right| ^2}{\rho _{\textrm{Si}} c_{\textrm{Si}}V_{\textrm{eff}}}\left( \frac{{\alpha _{\textrm{abs}}} c_0}{n_0}+\frac{c_0^2\beta _2\left| u \right| ^2}{n_0^2V_{\textrm{TPA}}} +\frac{\sigma _{\textrm{FCA}} N c_0}{n_0}\right) -\frac{\Delta T}{\tau _{\textrm{th}}}. \end{aligned}$$

Here, $U=\left| u\right| ^2$ is the mode energy, $\omega _L$ is the angular frequency of the input laser light, $\omega _0$ is the resonance angular frequency, $c_0$ is the speed of light in vacuum, $n_0$ is the group index of the waveguide, ${n_2 c_0}\left| u \right| ^2/{(n_0 V_{\textrm{Kerr}})}$ is the index change due to the Kerr effect with $n_2$ the Kerr coefficient, $V_{\textrm{Kerr}}=A_{\textrm{Kerr}}L$ the Kerr nonlinear volume, $A_{\textrm{Kerr}}$ the effective cross-section of the waveguide for the Kerr effect, and *L* the circumference of the MRR. The index change due to the FCD effect is introduced to the equations by the $\sigma _{\mathrm {r_1}}N + \sigma _{\mathrm {r_2}}N^{0.8}$ term where $\sigma _{\mathrm {r_1}}=8.8\times 10^{-28} \, m^3$ and $\sigma _{\mathrm {r_2}}=1.35\times 10^{-22} \,m^{2.4}$ relate the refractive index change to the electron and hole densities, respectively^23^. The index change due to the thermo-optic effect is given by $k_{\theta } \Delta T$ with $k_{\theta }$ the TO coefficient of silicon, and $\Delta T$ the temperature change of the ring resonator. The total linear loss (LL) of the MRR is $\alpha =\alpha _{ring}+\alpha _{c}$ where $\alpha _{ring}=\alpha _{abs}+\alpha _{rad}+\alpha _{sca}$ is the linear loss of the ring composed of three parts with $\alpha _{abs}$ the linear absorption loss which contributes to the heat generation and temperature increase in the silicon MRR, $\alpha _{rad}$ the radiation loss due to the coupling of light to the higher-order modes of the waveguide, and $\alpha _{sca}$ the scattering loss. $\alpha _{c}$ is the coupling loss. The nonlinear loss due to the TPA is $\beta _2 c_0 \left| u\right| ^2/({n_0 V_{\textrm{TPA}}})$ with $\beta _2$ the TPA coefficient and $V_{\textrm{TPA}}=V_{\textrm{Kerr}}$ the TPA volume. The FCA loss is ${\sigma _{\textrm{FCA}}N}$ with $\sigma _{\textrm{FCA}}$ the FCA coefficient. $\Gamma _c$ is the coupling coefficient between the straight waveguide and the ring waveguide given by $\Gamma _c=c\alpha _c/n_0$ and $s_{\textrm{in}}(t)$ is the power wave amplitude for the incident light in the waveguide so that the $|s_{\textrm{in}}(t)|^2$ gives the power carried by the incident light. In (2), Kerr effect, FCD, and TO effect contribute to the resonance shift, and the linear loss, TPA, and FCA affect the decay rate of the complex mode amplitude. $V_{\textrm{FCA}}=A_{\textrm{FCA}}L$ is the FCA volume, with $A_{\textrm{FCA}}$ the effective cross section of the FCA effect, $\hbar$ is the reduced Planck’s constant, and $\tau _{\textrm{car}}$ is the free carrier lifetime. In (3), free carrier’s generation is related to the TPA effect and their recombination lifetime is assumed constant ($\tau _{\textrm{car}}$). $\rho _{\textrm{Si}}$ and $c_{\textrm{Si}}$ are the density and the specific heat capacity of silicon, respectively, $V_{\textrm{eff}}=A_{\textrm{eff}}L$ is the effective volume of the silicon waveguide with $A_{\textrm{eff}}$ the effective cross-section of the waveguide^22^, and $\tau _{\textrm{th}}$ is the thermal decay time. In (4), the absorption part of the linear loss along with the TPA, and FCA nonlinear losses are the three sources for temperature increase and we assume that the temperature decays with a single decay time for simplicity^24^. Given the large differences in the magnitudes of the complex mode amplitude, free carrier density, and temperature change, we employ a normalized set of equations that uses dimensionless parameters to facilitate the numerical analysis^22^. The output power is $|s_{\textrm{out}}(t)|^2$ with $s_{\textrm{out}}(t)$ the complex amplitude of the output light $s_{\textrm{out}}(t)=\sqrt{P_\textrm{in}}-\sqrt{\Gamma _\textrm{c}} u(t)$.

In our calculations, we assume a group index of $n_0$= 4.1 and a loss of 2 dB/cm for the silicon waveguides. Values for the other parameters of the nonlinear model are summarized in Table S1.

**Fig. 2:**
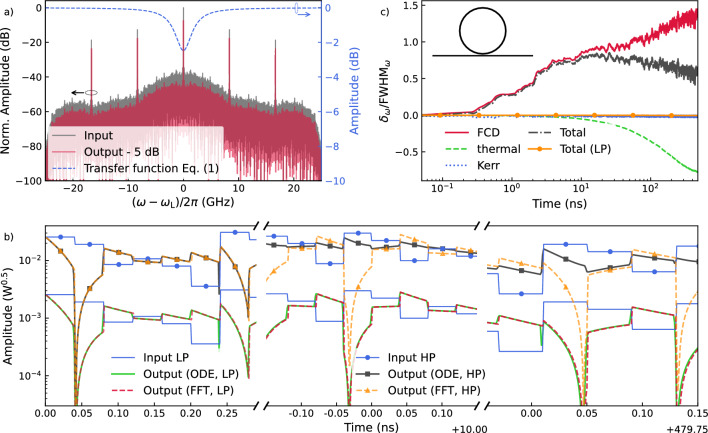
Linear and nonlinear responses of an all-pass ring resonator as part of a time-delay photonic reservoir. (**a**) Frequency spectra of an AM-modulated signal masked by a 3-element mask sequence (gray), the transfer function of a typical all-pass ring resonator (blue, left axis), and the output spectra (red, shifted vertically ($-5\, \textrm{dB}$) for better visibility). (**b**) Comparing the time responses of the ring resonator for the low-power (LP) and high-power (HP) cases using both linear and nonlinear analyses. In the LP regime, the models’ predictions are in good agreement, but as expected, they predict different behavior for high levels of input power (*e.g.* HP case). (**c**) Normalized frequency detuning ($\delta _\omega /FWHM_\omega ;\ FWHM_\omega \approx 2.4\, \textrm{GHz}$) vs. time (note the logarithmic scale) showing time-scales and strengths for FCD/FCA, TO, and Kerr effect when the ring is excited by a relatively high-power (HP) optical input signal ($P_{in}=1\,\text {mW}$). For the LP case ($P_{in}=10\,\mu \text {W}$), the total frequency detuning is always negligible. Inset shows a schematic of an all-pass ring resonator coupled to a nearby waveguide.

We now compare the linear solution with results from the nonlinear model in a silicon ring resonator to understand the input power levels for which the linear model correctly predicts the system’s response. In Fig. 2, the response of the linear and nonlinear model for an over-coupled all-pass ring resonator with a radius of 10 $\mu$m and a loaded quality factor of $Q_{l}=8.15\times 10^4$ ($FWHM_\omega \approx 2.4 \, \textrm{GHz}$) are presented. Here, the laser is centered around $\lambda _\textrm{L}=1550\, \textrm{nm}$ and we assume a normalized detuning of $\delta _\omega /\textrm{FWHM}_\omega =0$ with FWHM$_\omega$ the full width at half maximum of the resonance. Figure 1a (i) shows the schematic of the studied system. The input is an intensity modulated laser with modulation signal being a pseudo-random sequence of length 4000 with a uniform distribution in the range between 0 and 1 which is multiplied element-wise by a periodic mask of length three (M = [0.9, 0.5, 0.1]) such that each input symbol is temporally expanded into three consecutive masked values implemented using a sample-and-hold scheme, where each element of the pseudo-random sequence is held constant over a fixed time interval $\tau$ = 40 ps. The maximum instantaneous power of light in the bus waveguide is kept at 1 mW (high power (HP)) or 10 $\mu$W (low power (LP)). In Fig. 2a, the normalized spectrum of the masked input light and the output light is demonstrated (left axis) around the center frequency of the laser. The amplitude of the transfer function of the all-pass MRR is also shown in this figure (right axis). The spectra exhibit peaks at the harmonics of the fundamental frequency of the masking signal, and its bandwidth is 25 GHz. Considering the initial detuning of zero, the signal spectrum is aligned with the resonance of the transfer function. Figure 2b shows the input and the output signals in the time domain, calculated using either the linear or nonlinear model at three different periods of time (left: $t\approx 0\, \textrm{ns}$, middle:$t\approx 10\, \textrm{ns}$, right:$t\approx 480\,\textrm{ns}$) for both the LP and the HP cases. The field amplitudes of the LP and HP cases differ by a factor of ten reflecting the difference in maximum power. Since the resonator is overcoupled, it charges quickly within a single bit duration. The interference between the input light and the light leaving the charged ring resonator causes sudden rises and falls in the output signal. For the LP input, both models are always in good agreement. For the HP input, the models align initially because it takes time for nonlinear processes to appear (2b, left panel). The FCD/FCA response becomes apparent around $t\approx 1\, \textrm{ns}$ (2b, middle panel) and the impact of the TO process appears at around $t\approx 100 \, \textrm{ns}$. Figure 2c demonstrates the induced detuning of the ring resonator due to the nonlinear effects in the MRR for the same LP and HP input sequences. For the LP case, the total detuning is always negligible, confirming that the linear model suffices for predicting the MRR’s response. For the HP case, the detuning is significant, primarily due to the FCD/FCA and TO effects. Figure 2c also highlights different time scales of the nonlinear effects for typical silicon MRRs: several nanoseconds for the FCD/FCA and tens of nanoseconds for the TO effect. Although the Kerr effect is instantaneous, it is not strong enough to significantly affect the response and is usually overshadowed by the slower FCD/FCA effect.

## Reservoir computing with a linear PIC

### All-pass ring resonator

Figure 1a shows a schematic of the proposed reservoir computer. We denote the input sequence to the reservoir by $X\left[ n\right]$, where *n* is an integer index. The input sequence is multiplied by a mask sequence of length $\mathcal {M}$ denoted by $M\left[ n\right]$ (with $M\left[ n\right] \in [0,1]$ and again *n* an integer index), and is then modulated onto a continuous wave laser by either phase modulation (PM) or amplitude modulation (AM). For an input sequence $X\left[ n\right]$ and a mask sequence $M\left[ n\right]$, the input signal to the modulator is $x(t) \times m(t)$ with *t* the time and $x\left( t\right) =X\left[ n\right]$ for (n−1)$\mathcal {M} \, \tau _{p}< t < n\mathcal {M}\, \, \tau _{p}$ for *n* ranging from 1 to the length of input sequence.

The mask signal *m*(*t*) is defined as $m\left( t\right) =M\left[ n\right]$ for $(n-1) \, \tau _{p}< t < \, n \, \tau _{p}$ with *n* ranging from 1 to $\mathcal {M}$, and is repeated by a periodicity of $\mathcal {M}\tau _p$. Figure 1b shows a typical input signal *x*(*t*) and its corresponding masked signal $x(t)\times m(t)$ assuming that mask sequence is (1, 0.5, 0). Masking the input signal can improve the performance of a time-delay reservoir computer, but long masks decrease the computation speed. The modulation and masking non-linearly transforms the input sequence $X\left[ n\right]$ ($X\left[ n\right] \in [0,1]$) into either the phase or amplitude of the mode field of the light exiting the modulator. We later show that for the computational tasks considered in this work, binary masking (($M\left[ n\right] \in \{0,1\}$)) does not sacrifice the performance. For AM modulation, the mode amplitude at the input of the PIC is $s_{\textrm{in}}(t)=\sqrt{P_{in}\,m(t)\times x(t)}$^10,13^, and for the case of PM modulation, it is $s_{\textrm{in}}(t)=\sqrt{P_{\textrm{in}}}\exp {\left( -{j}0.6\pi x\left( t\right) \times m\left( t\right) \right) }$^25,26^. In each case, the maximum power of the modulated signal is $P_{\textrm{in}}$. At low powers, which are the focus in this work, the complex mode amplitude at the output of the PIC $s_{\textrm{out}}(t)$ is a linear transform of the input $s_{\textrm{in}}(t)$. In this case, delay elements in the photonic integrated circuit interfere with the input wave and its delayed copies using a Mach-Zehnder interferometer. A ring resonator or other passive PIC components can also act as an amplitude or phase filters. After the light passes through the PIC, it is detected by a photodetector whose output is proportional to the intensity of the mode amplitude at the output ($\left| s_{\textrm{out}}(t)\right| ^2$). This is another source of nonlinearity in addition to the modulation nonlinearity. The photodetected signal contains a nonlinear transform of the input signal and it is employed in a linear readout layer which can be trained to predict the desired output. A photonic reservoir configuration may^10,13^ or may not^27^ require digital memory; in the latter case, costly electronics are unnecessary. Figure 1c shows a target signal *y*(*t*) corresponding to the target sequence *Y*[*n*] (which is in this example is a non-linear autoregressive moving average (NARMA) task^28^) and a typical predicted signal $\hat{y}(t)$ (corresponding to $\hat{Y}[n]$) generated by the linear readout layer. The number of the samples used in the linear regression ($\mathcal {S}$) determines dimensionality of the output layer. The photodetector provides nonlinear functionality because its output is proportional to the intensity of the input light which may contain many interferences between delayed copies of the signal. Hence, if the detector bandwidth is large enough, the dimensionality of the reservoir may be increased by oversampling the photodetector signal.

We now briefly discuss the procedure to determine the parameters of the proposed optical time-delay reservoir computer. The shortest duration of the pulses $\tau _{pulse}$ that can be generated by the modulator is dictated by the available technology. Here we assume a pulse duration of $\tau _{pulse}=40 \, \textrm{ps}$ that is readily available in silicon photonics^29^. The nonlinear effects in a silicon MRR are usually very slow (FCA/FCD and TO effects) compared to available modulation speeds. The only exception is the Kerr effect. In spite of its speed, as shown in Fig. 2c this effect is weaker than FCD/FCA and TO effects and usually it is not possible to access a strong Kerr effect due to the presence of other nonlinear effects in silicon which are stronger and slower at the same time. Reducing free-carrier lifetime in silicon by applying a bias to the nano-waveguides^30^ can affect the balance between different nonlinear effects and potentially improve the performance of a photonic reservoir computer^12,31^. Another approach is based on using long mask sequences along with short input pulses (tens of picoseconds) to simultaneously increase the dimensionality of the reservoir and employ the FCD/FCA nonlinear effects^10,11,13^. However, for constant pulse duration, increasing the length of the mask sequence $\mathcal {M}$ will decrease the calculation speed by a factor of $1/\mathcal {M}$. Moreover, for a specific modulation rate and in the absence of slow nonlinear effects, using a longer mask may require longer and even off-chip delay lines so that the inputs at different time steps can interfere. This is because a longer mask sequence will prolong the time required to modulate a single entry from the input data. Consequently, the reservoir would require a longer delay line for efficient interaction between different entries of the input data. But a longer delay line may deteriorate the performance due to the losses in the larger feedback arm, and add complexity to the design. Since long delays are not easily achievable on a PIC, it will require more complex off-chip delay lines that may compromise the stability of the reservoir^11^. By assessing the reservoir’s performance at different input power levels, we find that a ring resonator in the nonlinear regime provides almost no major improvement in the accuracy of the NARMA-10 prediction task confirming previous observations^10,11,13^. Hence, here we limit the input power level to very small values (*e.g.* less than 10 $\mu$W) to eliminate any nonlinear effect in silicon and will show that the modulation and detection processes along with interferences present in a PIC can be effectively exploited for high-performance reservoir computation. In this approach, computation speed will no longer depend on the time scales dictated by free carrier dynamics or the thermo-optic effect. There are multiple metrics for assessing the performance of a reservoir. An important property of a reservoir is its capacity to recall simple linear or nonlinear transforms of its input at previous time steps^32^. To calculate the linear memory capacity (MC) one should train the reservoir so that its output mirrors the input at *k* time steps earlier for a varying number of timestep delays (*k*). The input to the reservoir in this analysis is derived from a uniform distribution. MC$_k$ is now defined as

5 $$\begin{aligned}&\text {MC}_k = \frac{\textrm{cov}^2(X[n-k],Y[n])}{\textrm{var}\left( X[n]\right) \textrm{var}\left( Y[n]\right) } , \end{aligned}$$

with $\textrm{var}(\cdot )$ and $\textrm{cov}(\cdot )$ the variance and covariance of the corresponding input sequences, respectively^10^. $\text {MC}_k$ quantifies how well the reservoir can recall its input value at *k* time steps earlier in the sequence, with $\text {MC}_k=1$ indicating perfect recall of that input value and $\text {MC}_k=0$ indicating no memory of it. The memory capacity is then calculated from

6 $$\begin{aligned} \text {MC} = \sum _{k=1}^{k_\textrm{max}} \textrm{MC}_k , \end{aligned}$$

with $k_\textrm{max}$ the largest number for which the $\textrm{MC}_k$ is calculated. A high MC is desirable for tasks where the output is dependent on inputs spanning multiple previous time steps. A high memory capacity is beneficial for tasks where the output at time t depends on input information from earlier time steps in the input sequence. To evaluate the potential of a reservoir computer for performing nonlinear computations, the reservoir is trained to predict a target signal which is a nonlinear transform of the input. The normalized mean squared error (NMSE) is then defined as:

7 $$\begin{aligned} \text {NMSE} = \frac{\sum { \left( Y[n] - \hat{Y}[n] \right) ^2}}{\sum \left( Y[n] - \bar{Y} \right) ^2} , \end{aligned}$$

where $Y[n]$ is the target sequence, $\hat{Y}[n]$ is the predicted sequence, $\bar{Y}$ is the mean of the target sequence.

**Fig. 3:**
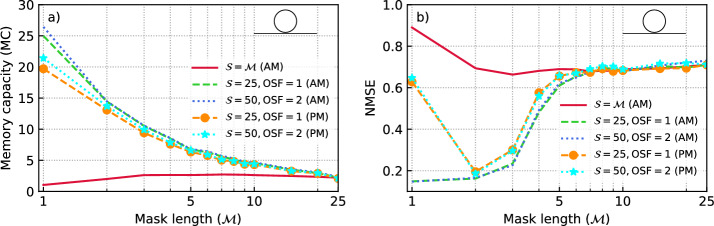
Impact of mask length on the performance of a photonic reservoir based on a single all-pass MRR. Memory capacity (MC) (**a**) and NMSE for the NARMA-10 prediction task (**b**) for different mask lengths $\mathcal {M}$, output layer dimensionality $\mathcal {S}$, and modulation scheme (amplitude modulation (AM) or phase modulation (PM)). For the $\mathcal {M}=\mathcal {S}$ case, both the nonlinear and memory functions are totally provided by the optical components. In other cases, the memory function is partially provided by the electronic linear readout layer which significantly improves the reservoir’s performance.

We investigate the impact of the mask length ($\mathcal {M}$), the detector’s oversampling factor (OSF), and the number of the photodetected samples used in linear regression ($\mathcal {S}$), on the performance of the reservoir for linear memory and NARMA10 prediction tasks. For these analyses, we consider a simple PIC composed of an all-pass ring resonator. The mask sequence is a pseudo-random vector of varying length $1\le \mathcal {M}\le 25$ with its element drawn from a uniform distribution $\mathcal {U}(0,1)$. In different analyses of this section, the mask sequence for each value of $\mathcal {M}$ is kept constant and we use a particle swarm optimization to find a combination of normalized detuning ($0\le \delta _\lambda /\textrm{FWHM}_\lambda \le 4$), and $0<2Q_{loaded}/Q_{int}<2$ which yields either a high MC for the linear memory task or a low NMSE for the NARMA10 task. We perform these calculations for different reservoir configurations. In all our simulations, we include the impact of the detector noise by considering a signal to noise ratio of $\textrm{SNR}=40 \mathrm {\ dB}$ at the detector. In Fig. 3a and b we demonstrate the MC for the linear memory task and NMSE for the NARMA10 task, respectively. First, dimensionality of the input to the linear readout layer equals to the mask length ($\mathcal {S}=\mathcal {M}$). It is observed that increasing $\mathcal {M}$ up to two or three will improve the performance of the reservoir for both tasks due to the increase of the input dimensionality. But any further increase does not provide any major improvement. It is possible to employ a longer mask (*e.g.* $\mathcal {M}=25$) along with an external feedback loops^10,11^, or serially coupled ring resonators^13^ to achieve a higher memory capacity and consequently a better NMSE for NARMA10 task, but this comes at the cost of lowering the computation speed and more complex photonic integrated circuit.

Another reservoir configuration can be based on storing data from previous steps in the linear readout layer as well and using it along with current data to perform the required computations. In other words, one can keep the dimensionality of the input to the linear readout layer constant (*e.g.* $\mathcal {S}=25,\text {or}\,50$) and use masks with shorter lengths (*e.g.* $\mathcal {M}=5$) so that the linear readout layer itself can complement the memory function of the PIC. In this cooperative-memory scheme, the combination of a modulator, PIC, and the detector provides the nonlinear interaction of the nodes that are less separated in time. This is later complemented by the linear interaction of the output nodes stored in linear readout layer which can be more distant in time. The reservoir configuration with a short mask length $\mathcal {M}$ does not require different hardware than the reservoir with ($\mathcal {S}=\mathcal {M}=25$), provided that the output dimensionality is kept identical ($\mathcal {S}=25$). However, it can offer a significantly higher computation speed because it uses a shorter mask. More importantly, it greatly reduces the stringent requirements on the delay lines and facilitates a practical realization. Figure 3 shows the MC and NMSE for the second reservoir configuration with $\mathcal {S}=25$ for both AM and PM modulation of the input data. For AM modulation, increasing $\mathcal {M}$ decreases the performance for both linear memory and the NARMA10 tasks. As expected, the memory capacity which is mainly provided by the regression unit is much larger than for the first RC configuration. The higher memory capacity is also reflected in the substantially better performance for the NARMA10 task. The situation is almost similar for the PM and the performance of the reservoir shows a similar trend as the AM. In the case of PM, the memory capacity remains significant -though slightly smaller than the AM case- because the interferences occurring in the PIC practically convert phase modulation into amplitude variations which can then be detected by the intensity detector. The other difference is that in the case of no mask ($\mathcal {M}=1$), NMSE for the PM is much larger than the AM. As a final configuration for the RC, we consider the case where the detector signal is over-sampled by a factor of two compared to the modulation rate ($\textrm{OSF}=2$). Furthermore, we double the dimensionality of the linear readout layer ($\mathcal {S}=50$). Our computations as summarized in Fig. 3 show that in this case, the memory capacity is slightly improved, but the performance of the RC for the NARMA10 task remains unchanged.

### MZI-MRR configuration

**Fig. 4:**
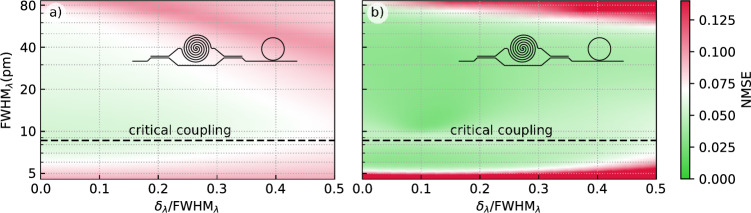
NMSE for the NARMA-10 prediction task, when the input is amplitude modulated (**a**) or phase modulated (**b**), for different values of normalized detuning and different resonator linewidth.

In the previous section we showed that the photonic reservoir based on a linear single MRR, despite its significantly simpler structure, achieves NMSE comparable to an MRR with external feedback for the NARMA-10 prediction task, provided that the reservoir’s parameters are optimally selected (*e.g.* $\mathcal {M}=1, \, \mathcal {S}=25$). The reservoir with a single MRR achieves such performance at considerably higher speeds than MRRs in the nonlinear regime^10^ because it employs no mask sequence while keeping the output layer complexity identical ($\mathcal {S}=25$). Both of these photonic reservoirs perform better than a shift register that contains the input data which achieves a minimum NMSE of 0.4^4^. We can further enhance the performance of our photonic reservoir by increasing interaction between the different nodes of the reservoir. Mach-Zehnder interferometers can provide linear transformation^33–36^ on their inputs. The transfer function of a Mach-Zehnder interferometer with a path-length difference *L* can be expressed as $T_{\textrm{MZI}}(\omega )=\tfrac{1}{2}\left( 1+e^{-\gamma (\omega )L}\right)$. Here we propose using an unbalanced MZI in conjunction with an all-pass ring resonator (Fig. 1a (ii)). By adjusting the delay difference between the arms of the MZI and the ratio of its input and output couplers, this configuration allows for tuning the interference between any two input nodes that are separated by a known time interval. An MZI with a large delay difference between its arms enables the interaction of input nodes that are far apart. On the other hand, an MRR whose memory capacity is related to its photon lifetime is used for interaction of the nodes that are less separated in time. The linear dynamics of the PIC are described by the transfer-function models of the MZI and MRR provided before, whereas nonlinear functionality at the photodetector and modulator are applied directly to the time-domain signals. We now investigate how the linear MZI-MRR-based reservoir computer performs in various computational tasks starting with the NARMA-10 prediction task. For this purpose, we consider symmetric 3-dB input and output couplers at the MZI’s ends to maximize the interaction of the nodes. Delay difference in MZM arms is set to $\tau _{\text {delay}}=9\mathcal {M}\tau _\text {p}$. For mask lengths $1\le \mathcal {M}\le 5$ the delay difference is $0.4 \text { ns}\le \tau _{\textrm{delay}}<2 \text { ns}$ which can be implemented on chip considering current available technologies^37^. To simplify the practical implementation of the masking process we assume a binary mask sequence with $M[n] \in \{0,1\}$ and examine all possible binary mask sequences to find the best performance. For each possible binary mask sequence, we try to find the optimum linewidth of the cavity ($\text {FWHM}_\lambda$) and its normalized detuning ($\delta _\lambda /\text {FWHM}_\lambda$) by running a particle swarm optimization (PSO). We explore the design space by running the PSO algorithm five times with randomly generated initial particle swarms. Each run was allowed a maximum of 400 iterations. In this analysis, the output layer dimensionality is $\mathcal {S}=50$ and we keep the ring radius and the waveguide properties as before. We do these calculations for both AM and PM modulations. For AM modulation with a modulation index of 1, the best NMSE is found once the mask sequence is $M=(1,0)$ which results in $\textrm{NMSE}=0.052$. If we remove the PIC from the photonic reservoir configuration of Fig. 1 and keep the other configuration unchanged, the performance of the system becomes similar to a linear shift register that contains the input sequence. It is known and our simulations confirm that such a reservoir provides a minimum NMSE of around 0.16^10^ for the NARMA-10 task. For PM modulation, the best NMSE is less than half of the AM case ($\textrm{NMSE}=0.024$) with the mask sequence $M=(1,0,0)$. With the PIC removed from the circuit, the NMSE for PM modulation is 1, as the intensity of the phase-modulated light detected by a photodetector is constant. Figure 4 shows the calculated NMSE as a function of cavity linewidth and detuning for AM (Fig. 4a) and PM (Fig. 4b) modulations when their specific optimized mask sequence are employed. The reservoir’s performance is best in a region around the critical coupling condition and slight or no detuning. In an experiment, the detuning can be controlled by adjusting the temperature of the chip or using a tunable laser^38,39^. The resonator linewidth, on the other hand, is controlled either by the gap between the bus waveguide and the ring resonator or electrically using a more sophisticated coupler design^40^.

Next, we investigate how MZI-MRR performs for other computing purposes. Similar analyses are performed for the Santa-Fe and Mackey-Glass prediction tasks with the same parameters as reported in previous studies^10,13^. Here we also keep the MZM parameters exactly the same as before and try different modulations, masks, detunings, and coupling conditions of the MRR to find a suitable combination. We study this scenario, so that the same PIC will be able to handle different computational tasks with adjustments made only to its operating point.

For the Mackey-Glass prediction task^10,13^ and in the presence of noise (SNR = 40 dB), the reservoir performs well for AM and PM modulation even when no mask sequence is applied. For AM modulation (modulation index of 1), we find NMSE = 0.0035, and for PM modulation NMSE = 0.002. Without PIC in the configuration, we find NMSE = 0.0035 for AM modulation, and NMSE = 1 for PM modulation. For this task, the computation speed is equal to the modulation speed (25 GHz).

For the Santa-Fe prediction task^10,13^, AM modulation and a binary mask of length 4 ($M=(1,1,0,1)$) we find $\textrm{NMSE}=0.06$. This can be improved to $\textrm{NMSE}=0.044$ when the modulation index is 0.5 and the mask is ($M=(0,1,1,0)$). Figure S1a shows the NMSE of Santa-Fe prediction tasks for different normalized detunings and coupling conditions. For PM modulation, the best performance we find is when the mask is $M=(1,0,1,1)$ that gives $\textrm{NMSE}=0.053$ (Fig. S1b). It is expected to achieve better performance if one tunes other parameters of the reservoirs as well. For example, an NMSE of 0.026 can be achieved with a mask $M=(1,0,1,1,1)$ if the sampling rate at the detector is doubled, without changing any other parameter of the reservoir. In the abscence of PIC, NMSE = 0.2 for AM modulation, and NMSE=1 for PM modulation. It is worth noting that the Santa-Fe dataset contains experimental noise, which suggests that the proposed reservoir computer can perform well in the presence of a noisy input.

Table 1 summarizes the performance of some of the most recent MRR-based photonic RCs. Size of the shift register in previous studies was between 25 and 200^10,13^ in different cases. In our case, we use a shift register of size of either 25 or 50. In all of these studies, the modulation and detection rates are 25 GHz. Without sacrificing the accuracy, the computation speed in this work is improved due to the use of a short mask and the proposed co-operating memory configuration. In case of the NARMA-10 prediction task, both the computation speed and the accuracy are significantly improved. For the Mackey-Glass and Santa-Fe tasks, the reservoir is highly accurate and performs comparable to the previous proposals.

**Table 1:** Recent MRR-based photonic reservoir computers.

Structure	NARMA-10		Mackey-Glass		Santa-Fe	
	NMSE	Speed (GHz)^*^	NMSE	Speed (GHZ)*	NMSE	Speed (GHz)*
MRR^13^	0.534	1	0.0137	1	0.038	1
SCMRRs^13^	0.156	1	0.0083	1	0.018	1
MRR+Feedback^10^	0.187	1	0.0014	1	0.02	1
MRR (AM)**	0.15	25				
MRR (PM)**	0.2	12.5				
MZI-MRR (AM)**	0.052	12.5	0.0035	25	0.044	6.25
MZI-MRR (PM)**	0.024	8.33	0.002	25	0.053	5

*Computation speed assuming that the modulation and detection rates are 25 GHz.

**This work.

## Optical signal equalization

This section studies the signal equalization capabilities of the MZI-MRR structure shown in Fig. 1a (ii) numerically in a simulated short-reach fiber-optic transmission^41,42^ and compared to the feed-forward equalizer (FFE) as an established digital signal processing. It is parameterized based on a recently published IEEE standard for 800 Gb/s Ethernet operation which suggests using wavelength-division multiplexing (WDM) in the O-band with 112Gbaud 4-level pulse-amplitude modulation (PAM)^43,44^. In this scenario, the RC has to mostly compensate linear impairments, namely accumulated chromatic dispersion of the optical fiber and chirp from an electro-absorption-modulated laser (EML).

For this simulation in the O-band, a zero dispersion wavelength (ZDW) of 1310 nm is assumed with a dispersion slope of 0.09 $\textrm{ps}/\textrm{nm}^2.\textrm{km}$. With a channel separation of 400 GHz as proposed in^41,42^, the impact of crosstalk is negligible. Therefore, we simulate a single channel that is most impaired by chromatic dispersion and modulation chirp to investigate the worst-case scenario. For this, a pseudo-random binary sequence (PRBS) with $2^{18}$ bits is modulated using PAM-4. This digital signal is shaped using a raised cosine function with a roll-off factor of 0.05 before being converted into an optical signal using an EML at 112Gbaud with a linewidth enhancement factor $\alpha _c=-0.5$ and a 3 dB bandwidth limitation of 50 GHz using a Butterworth filter. Before and after the signal is filtered using a Gaussian filter with a cut-off frequency of 400 GHz to emulate the effect of (de)multiplexing. Next, the signal is coupled to the reservoir considering 3 dB coupling losses. The output of the reservoir is amplified by a semiconductor optical amplifier (SOA) with a noise figure $n_f = {6}\ {dB}$ to compare it to conventional signal processing based on feed-forward equalization at the same received optical power (ROP). Detection is implemented using a non-ideal photodiode with a cut-off frequency of 80 GHz. Furthermore, a dark current of 2$\times 10^{-8}$A, shot and thermal noise are added to the received signal before it is resampled to 224 GSa/s. The readout layer is implemented as an finite impulse response filter with an identical number of taps as the baseline FFE. Instead of a conventional least-mean squares algorithm, the readout layer is trained to reconstruct the transmitted symbol using linear regression with Tikhonov regularization with $\alpha _{R}=1$ based on singular value decomposition. Both the baseline FFE and the readout layer are trained using 2048 known training symbols. Therefore, the computational complexity and memory requirements in the digital domain using RC are identical to the FFE baseline, except for the training procedure.

The reservoir’s parameters are optimized using random search with 1000 iterations at an accumulated dispersion of − 16 ps/nm and an ROP of − 8 dBm. The normalized detuning is drawn from $d_d \sim \mathcal {N}(0, 10)$, while the MZI delay is drawn with a discrete uniform probability distribution containing half increments up to 10 of the symbol duration of $\approx {4.4}\ {ps}$. The coupling factor for each branch is drawn from $\mathcal {U}(0,1)$ and the Q-factor from $\mathcal {U}(0.025,.975)\times Q_{int}$, ranging from strong over-coupling to extreme under-coupling. The radius of the MRR is kept constant at 10 µm. The best configuration identified in this process is shown in Supplementary Table S2.

**Fig. 5:**
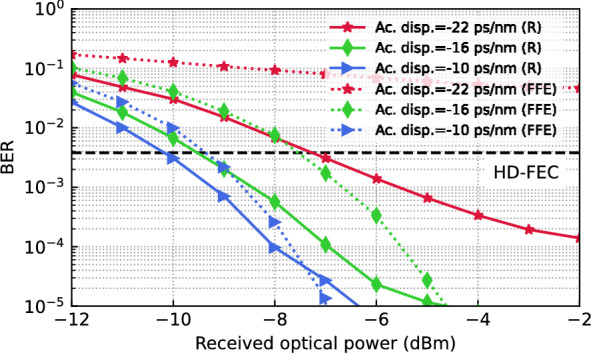
Photonic signal equalization using MZI-MRR. Bit error rate (BER) versus received optical power (ROP) for different accumulated dispersion when either a photonic reservoir (R) or an FFE is employed for equalization. Parameters of the photonic reservoir are optimized for an accumulated dispersion of -16 ps/nm and an ROP of − 8 dBm.

As shown in Fig. 5, the RC is able to reduce the BER compared to an FFE of identical complexity (with 25 first order coefficients) as the readout. Even for higher or lower amounts of dispersion, a clear advantage of using the RC can be seen, especially for ROPs above − 8 dBm where the system is not as severely affected by noise. Even though the RC was optimized for a single amount of accumulated dispersion, it is able to compensate − 20 ps/nm of accumulated dispersion, significantly better than the FFE. At lower amounts of accumulated dispersion, only a small improvement can be seen at lower ROPs. Considering the most impaired channel of the 800G LR-4 proposal with a center wavelength of 1306.57 nm, this improvement translates to an increase in transmission reach of 15 km, since, depending on ROP, up to − 5.5 ps/nm of additional accumulated dispersion can be tolerated before reaching the HD-FEC limit. This concept can be applied to WDM transmission, where a dedicated MZI-MRR is required for each channel after demultiplexing.

## Conclusions

This study shows that a PIC composed of an MRR in the linear regime fed by a MZI enables high-speed reservoir computation. Not limited by the free carrier lifetime or other comparably slow nonlinear processes, such a reservoir relies merely on the modulation detection nonlinearity and its calculation speed is only limited by the modulator/detector bandwidth. The proposed reservoir configuration improves the memory capacity by shortening the mask length while keeping the size of the shift register in the electronic linear readout layer constant. In this configuration, the electronic output layer can consider photodetected signals of the previous time steps as well. The MZI-MRR structure proposed in this work, along with the cooperative reservoir configuration, enables high-accuracy and high-speed computation for all different tasks explored *i.e.* NARMA-10, Santa-Fe, and Mackey-Glass. Our simulations show that computation speeds from 8.33 to 25 GHz for the time-series prediction tasks can be possible. Also, the accuracy (NMSE) is improved almost ten times for the NARMA-10 prediction task and it is comparable to the previous studies for the Mackey-Glass and Santa-Fe tasks. The computation speed in this configuration is higher than in previous studies without employing any different hardware, but by tailoring the masking, modulation, and linear response of the PIC. Here, we find that phase modulation can perform better for some computational tasks. A more detailed study of the modulation/detection schemes may reveal ways to improve the nonlinear function of the reservoir and the computation performance. Since the reservoir computer proposed in this research does not depend on free-carrier nonlinearity, increasing the computation speed up to 110 GHz is currently accessible considering the state-of-the-art integrated optical modulators. This will simplify the design of the PIC as well because delay lines with shorter lengths and ring resonators with lower Q-factors will be required. Furthermore, the MZI-MRR configuration proposed here can be extended to an array with elements that are different in their delay lines and MRRs, in order to perform more complex computations. In such a scenario, the electronic storage of the data along with the rich and diverse interference of the optical signals in the PIC will contribute to high-speed and accurate photonic computation.

## Supplementary Information

Below is the link to the electronic supplementary material.

Supplementary Material 1

## Data Availability

The datasets used during the current study are available from the corresponding author upon reasonable request.
