# Supplementary material for: A cascaded interferometer-microresonator structure for photonic reservoir computing
Source: Sci Rep. 2026 Feb 14;16:6492. doi: 10.1038/s41598-026-39410-w (PMC12909825; doi:10.1038/s41598-026-39410-w)
Supplement: Supplementary file 1 — Supplementary Material 1 [file 41598_2026_39410_MOESM1_ESM.pdf]

A Cascaded Interferometer-Microresonator Structure for Photonic Reservoir Computing

Amideddin Mataji-Kojouri[1]*, Sebastian Kühl[2],

Mohammad Seifi Laleh[2], Chandan Upadhyay[1], Stephan Pachnicke[2], Kambiz Jamshidi[1]*

[1]*Integrated Photonic Devices Group, Chair of RF and Photonics Engineering, TU Dresden, Helmholzstr. 18, Dresden, 01069, Germany.

[2]Chair of Communications, Kiel University, Kaiserstr. 2, Kiel, 24143, Germany.

*Corresponding authors. E-mails:

amideddin.mataji_kojouri@tu-dresden.de;

kambiz.jamshidi@tu-dresden.de;

Contributing authors: sk@tf.uni-kiel.de; mola@tf.uni-kiel.de;

Chandan.upadhyay@tu-dresden.de; stephan.pachnicke@tf.uni-kiel.de;

Supplementary Information

Table S1. Parameters of the nonlinear model

Parameter	Value	Unit
n_0	4.1	
α_{ring}	46 ($L = 2\ dB/cm$)	1/m
η_{lin}	0.4	
R	10	μm
n_2	4.5×10^{-18}	m^2/W
β_2	7.5×10^{-12}	m/W
σ_{FCA}	1.45×10^{-21}	m^2
A_{eff}	2.04×10^{-13}	m^2
A_{TPA}	1.289×10^{-13}	m^2
A_{FCA}	1.16×10^{-13}	m^2
ρ_{Si}	2.329×10^3	kg/m^3
c_{Si}	713	J/(kg K)
k_θ	1.86×10^{-4}	K^{-1}

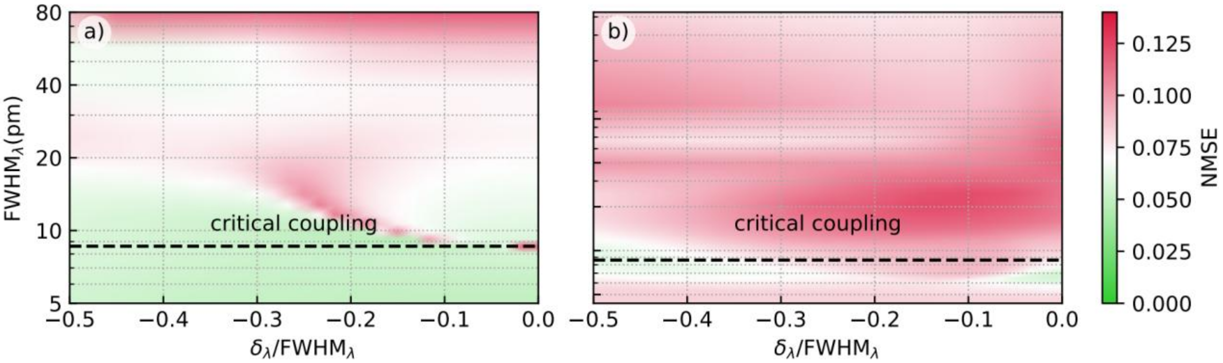

Fig. S1 NMSE for Santa-Fe prediction task, when input is amplitude modulated (a) or phase modulated (b), for different values of normalized detuning and resonator linewidth controlled by the coupling strength.

Table S2. Parameters of the optimized PIC for optical signal equalization

Parameter	Value	Unit
MZI delay difference	4.46	ps
Q/Q_{int}	0.69	-
MZI input coupling ratio	0.47	-
MZI output coupling ratio	0.116	-
Normalized detuning	0.843	-
